# Audiological Outcome of Classical Adenoidectomy versus Endoscopically-Assisted Adenoidectomy using a Microdebrider

**Published:** 2016-01

**Authors:** Vanita Sarin, Vanika Anand, Bhanu Bhardwaj

**Affiliations:** 1 *Department of Otorhinolaryngology, Sri Guru Ram Das Institute of Medical Sciences & Research, Amritsar,Punjab, India.*; 2 *Department of Otorhinolaryngology, Christian Medical College, Ludhiana,Punjab, India.*

**Keywords:** Adenoidectomy, Audiological, Endoscopic, Microdebrider, Tympanometry.

## Abstract

**Introduction:**

The aim of this study was to evaluate audiological outcomes following adenoidectomy by the classical method and by endoscopically-assisted adenoidectomy using a powered instrument (microdebrider).

**Materials and Methods:**

This study was conducted in a tertiary care center. It included 40 patients divided into two equal groups of 20 each. Group-A patients underwent classical adenoidectomy, while Group-B patients were subjected to endoscopically-assisted adenoidectomy using a microdebrider. Hearing outcome was measured by post-operative pure-tone audiometry and tympanometry.

**Results:**

The post-operative average air-bone gap (ABG) was reduced from 19.6 dB to 11.8 dB in Group A and from 17.6 dB to 8.7 dB in Group B (P=0.010). There was reversal of tympanometric curves from type-B and type-C to type-A in 55% of the patients in Group A, while type-A curve was seen in 90% cases in Group B in the post-operative period.

**Conclusion:**

Audiological outcomes of endoscopically-assisted adenoidectomy using a microdebrider were superior compared with classical adenoidectomy.

## Introduction

With the exception of the common cold, otitis media is the most common disorder for which children and their families seek pediatric care. Otitis media with effusion (OME) is the most common cause of hearing loss in children today. It is characterized by the collection of serous or mucous fluid in the middle ear behind an intact tympanic membrane during an inflammatory process, and a lack of acute signs and symptoms of infection (1). OME results from alterations in the mucociliary system within the middle ear cleft where serous/ mucoid fluid accumulates in association with negative pressure. This pressure change is almost invariably caused by a malfunction of the Eustachian tube. The main etiology of OME is Eustachian tube dysfunction, as the Eustachian tube is the only route of infection to the middle ear. Hypertrophied adenoid tissue can lead to the mechanical and/or inflammatory obstruction of the nasopharyngeal ostium of the auditory tube, leading to Eustachian tube dysfunction, which represents one of the most frequent otological pathologies, and the starting point for almost all acute or chronic otologic inflammatory processes and their consequences (2). It can cause recurrent otalgia, impaired hearing (hypoacusis) and delayed defective speech. Bluestone and Doyle attributed three major functions to the Eustachian tube– ventilation, clearance and protection of the middle ear (3). Any disturbance of these functions can lead to Eustachian tube dysfunction.

The most important Eustachian tube function and the first to be affected by hypertrophied adenoids is ventilation of the middle ear. 

Clinical audiometric and tympanometric assessment may be used for screening and diagnosis. However, the gold standard test for evaluating the middle ear pressure, air volume and tympanic compliance is tympanometry. Treatment varies widely and is dependent on the duration and severity of the condition. Traditional adenoidectomy is performed using an adenoid curette and, as known, this method does not always completely remove the adenoid tissue. This method showed effective tissue removal in only 30% of cases (4).

Moreover, excessive removal of adenoid tissue by this method may provoke damage to the pharyngeal muscles, posterior choana, Eustachian tube orifice or other structures. As a result, several complications may result from a traditional adenoidectomy (5). In the 1990s, the advent of endoscopic sinus surgery popularized the use of intranasal endoscopes, and the endoscopic adenoid- ectomy became the natural evolution of conventional adenoidectomy, allowing direct visualization throughout the procedure (6,7). Post-operative complications such as velopharyngeal insufficiency, tubaric stenosis and nasopharyngeal stenosis are rare. By using this technique, the adenoid remnants along the superior portion of the nasopharynx, the choanae, and the peritubal region can be visualized and thus removed completely; moreover, the likelihood of damage to the adjacent areas is reduced and the hemorrhage can be effectively controlled by direct identification of the bleeding points (6,7).

Another advancement in this field was the use of microdebriders; usage of which showed a lower incidence of complications. In 2002, a study by Rodriguez et al. showed no evidence of long-term complications, including significant blood loss, in over 1,000 procedures carried out using the powered-assisted instrument (8). This prospective study was conducted to compare the audiological outcome of adenoidectomy using the classical method versus endoscopically-assisted adenoidectomy using powered instruments (microdebriders).

## Materials and Methods

This prospective study was conducted in atertiary care center. The study included 40 pediatric cases (age, 3–14 years), having symptoms suggestive of adenoid hypertrophy and OME, confirmed by pure-tone audiometry (PTA) and tympanometry. Children aged <3 years and >14 years were excluded. Children with craniofacial deformities, deranged coagulation profile, or cleft palate were also excluded. All selected cases were thoroughly examined. An X-ray of the nasopharynx (lateral view) was performed. Grading of the adenoid hypertrophy was conducted according to the grading system devised by Fujioka M et al. (9). The assessment of adenoidal-nasopharyngeal (AN) ratio was performed according to the method of Fujioka M et al. (9). 

 A preoperative diagnostic endoscopy for grading of the adenoid hypertrophy was performed according to the scale devised by Clemens and MacMurray (10):

- Grade I: Adenoid tissue filling 1/3 the vertical height of choana
- Grade II: Adenoid tissue filling 2/3 the vertical height of choana
- Grade III: Adenoid tissue filling from 2/3 to nearly all but not completely filling the choana
- Grade IV: Complete choanal obstruction.

All patients were randomized into one of two groups. Group A consisted of 20 cases of adenoid hypertrophy with OME undergoing conventional curettage adeno- idectomy; Group B consisted of 20 cases of adenoid hypertrophy with OME undergoing endoscopically- assisted adenoidectomy with microdebrider. Postoperatively, on follow-up 12 weeks after adenoidectomy, all 40 patients underwent PTA and impedance tympa- nometry.

## Results

In our study, 70% of cases were male and 30% were female. The mean age of presentation in Group A was 8.68 years and in Group B, was 7.70 years. 

**Fig 1:**
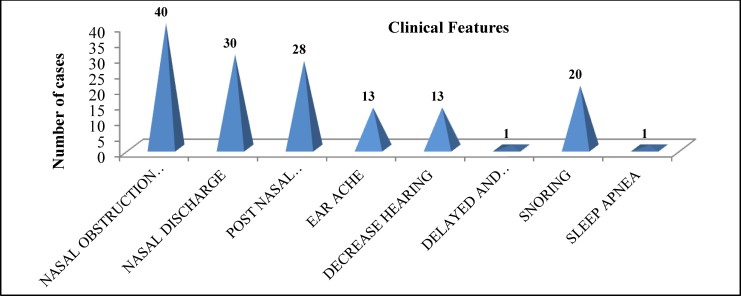
Clinical features

Nasal obstruction was the most common complaint, with an incidence of 100%, followed by nasal discharge (75%), post-nasal discharge (70%) and snoring (50%). Earache and impaired hearing both had an incidence of 32.5%. Delayed defective speech and sleep apnea both showed an incidence of 2.5% each (Fig.1).

Adenoid facies was the most consistent and constant feature on general examination in our study, accounting for 75% incidence. Oral cavity examination revealed that 92.5% cases presented with an open mouth and had a high arched palate. Radiological evaluation of the nasopharynx showed that in all cases the AN ratio fell between 0.73–0.99. On nasal endoscopy, Grade III adenoid hypertrophy was the most common in both groups (Table.1).

**Table 1:** Grading of adenoid hypertrophy by nasal endoscopy (Clemens & McMurray Grading

**Grades**	** Group A **		**Group B**	
	No. Patients	Percentage	No. Patients	Percentage
I				
II	3	15%	2	10%
III	13	65%	13	65%
IV	4	20%	5	25%

The preoperative PTA of Group a showed that 60% patients had an air-bone gap (ABG) within 11–20 dB followed by an ABG within 20–30 dB in 25% of patients (Table.2).

**Table 2:** Pre- and post-operative ABG in Group A

**ABG**	**Pre-op**		**Post-op**	
	No. Patients	Percentage	No. Patients	Percentage
<10Db	1	5%	12	60%
11-20Db	12	60%	7	35%
21-30dB	5	25%	1	5%
30-40dB	2	10%		

Preoperative tympanograms revealed that out of 20 cases in this group, type-C curve was present in 70% while type-B curve was seen in 30% cases (Table. 3).

**Table 3:** Pre- and post-operative tympanogram in Group A

**Tympanogram**	**Pre-op**		**Post-op**	
	No. Patients	Percentage	No. Patients	Percentage
Type-A			11	55%
Type-B	6	30%	1	5%
Type-C	14	70%	8	40%

Post-operative PTA of Group A revealed that the ABG was reduced to <10 dB in 60% of cases, while it was reduced to <20 dB in 90% of cases.Only 5% of cases had an ABG within 20–30 dB postoperatively (Table.2). Thus, there was a reduction in average ABG in this group from 19.6 dB to 11.8 dB. The post-operative tympanogram revealed that approximately 55% cases out of a total of 20 in this group showed a type-A curve followed by type-B and type-C curves in 5% and 4%, respectively (Table. 3). Preoperative PTA of Group B showed an ABG within 11–20 dB in 75% of cases followed by an ABG within 21–30 dB (10%) and 31–40 dB (10%) (Table.4).

**Table 4:** Pre- and post-operative ABG in Group B

**ABG**	**Pre-op**		**Post-op**	
	No. Patients	Percentage	No. Patients	Percentage
<10Db	1	5%	18	90%
11-20dB	15	75%	2	10%
21-30dB	2	10%		
30-40dB	2	10%		

Preoperative tympanograms revealed that of the 20 cases in this group 85% had a type-C curve while the remaining 15% had a type-B curve (Table. 5).

Post-operative PTA of this group revealed that the ABG was reduced to <10 dB in 90% of cases and to <20 dB in all cases (Table.3). Thus, the average ABG in this group was reduced from 17.6 dB to 8.7 dB in the post-operative period. Post-operative tympano- gram showed that approximately 90% cases had a type-A curve followed by type-C (5%) and type-B curve (5%) (Table.5). Statistical comparison of the two groups showed a statistically significant difference (P=0.010).

**Table 5:** Pre and post-operative tympanogram in Group B

**Tympanogram**	**Pre-op**		**Post-op**	
	No. Patients	Percentage	No. Patients	Percentage
Type-A			18	90%
Type-B	3	15%	1	5%
Type-C	17	85%	1	5%

## Discussion

With reference to national and international studies, the mean age of presentation in our study corresponds well with other studies. Adenoid hypertrophy can present with varied symptoms ranging from nasal obstruction, nasal discharge, post-nasal discharge, snoring, earache, impaired hearing, and sleep apnea to delayed defective speech (11,12). 

Hypertrophied adenoid tissue causing habitual mouth breathing may result in dentoskeletal malocclusions. These dentofacial changes were termed as ‘adenoid facies’’ by Tomes in 1872, with the belief that enlarged adenoids were the principal cause of airway obstruction leading to noticeable dentofacial changes (13). In our study, adenoid facies is one of the most consistent and constant features. Eustachian tube orifices are in close proximity to the adenoidal pad. Eustachian tube dysfunction and chronic rhino-adenoiditis represent two related pathologies, with respect to the fact that the obstruction and the inflammation that appear secondary to adenoid hypertrophy can lead to auditory tube dysfunction. Within the pediatric population, rhinopharynx (nasopharynx) lymphoid tissue hypertrophy is the primary cause of Eustachian tube dysfunction and its complications. The otoscopic examination of 40 cases in our study revealed that 62% had retracted tympanic membrane and 35% had OME (air bubbles with a dull, lusterless tympanic membrane). This is supported by a study of Sarafoleanu et al. published in 2010 which showed the implications of adenoid tissue hypertrophy in the genesis of Eustachian tube dysfunction (2). On otoendoscopy, 29% patients had retracted tympanic membrane, while air bubbles with a yellow tympanic membrane suggestive of middle ear effusion were seen in 49% of patients. The remaining 22% had a normal tympanic membrane (2). Similarly, a cross-sectional study undertaken by Caylan et al. in 2006 also concluded that mechanical obstruction of the nasopharyngeal opening of the Eustachian tube by adenoid hypertrophy causes OME (14).

The clinical manifestations of adenoiditis may be readily remedied by removal of the obstructive hypertrophied adenoid tissue to restore airway patency. The dissatisfaction over the widely used conventional curettage adenoidectomy has prompted the use of endoscopic assisted powered shaver adenoidectomy with microbebrider (15). Murray et al. Rodigruez et al. Costantini et al. Proved that endoscopic assisted powered shaver adenoidectomy is more effective in cleaning adenoid tissue under direct visualization; thus requiring less operating time, causing less blood loss, and providing more complete removal of the adenoid tissue and less post-operative pain (10,16,17).

Pre- and post-operative nasal endoscopy has a significant role in assessing the completeness of removal of the hypertrophied adenoid tissue, especially in the areas of the Eustachian tube orifice and intranasal protrusions, and in assessing the intraoperative trauma caused by the operative technique (18). According to literature, regrowth of the residual lymphoid tissue left as a result of blind removal causes significant recurrence of symptoms (19).

Tympanometry is of enormous use in evaluating the middle ear pressure, air volume, and tympanic compliance. It was also used in our study for the diagnosis and evaluation of the outcome of the two surgical techniques. Our study revealed that adenoidectomy does improve the tympanometric findings. It shows that Group-B patients showed greater reversal of curves from type-B and type-C to type-A as compared with Group A; thus proving that the endoscopically-assisted adenoidectomy with microdebrider is superior. 

Although no systematic review of the literature has been performed, a number of similar studies have been reported. For example, a study by Sarafoleanu et al. in 2010 revealed a type-B curve in 41% of cases, compared with type-A in 22% and type-C curve in 37% of cases. Re-evaluation performed after 4 weeks of surgery (classical adenoidectomy) in their study also documented very good relief of disease on subjective as well as objective evaluation (2). Another study by Mori et al. in 1980 also observed a type-B tympanogram in 50% of cases preoperatively with post-operative conversion to type-A (20). By using endoscopic assisted adenoidectomy with a microdebrider, the adenoid remnants along the superior portion of the nasopharynx, the choanae and the peritubal region, can be clearly visualized and thus removed completely. Moreover, the likelihood of damage to the Eustachian tube and/or to the pharyngeal muscles is reduced, thereby reducing the post-operative scarring. Hemorrhage can also be effectively controlled by direct identification of the bleeding points (6,7). 

PTA provides some assessment of the severity of the disease and can be used to monitor progress and the effects of treatment. In our present study, it was observed that there was improvement in the average ABG in Group A from 19.6 dB to 11.8 dB, while in Group B the average ABG was reduced from 17.6 dB to 8.7 dB (P=0.010), showing that endoscopically-assisted adenoidectomy with microdebrider is superior; although there was no review to support this. Thus, adenoidectomy by either method in children having hypertrophied adenoids with OME not only relieves Eustachian tube obstruction but also removes a source of infection. This leads to clearance of middle ear effusion and improvement in post-operative hearing, and this study supports that endoscopically-assisted adenoidectomy with a microdebrider is superior to classical adenoidectomy with respect to post-operative otological outcomes.

## Conclusion

Adenoidectomy is a beneficial procedure for hearing improvement and for the eradication of OME. Endoscopically-assisted adenoidectomy with powered instruments (microdebrider) has shown improved results as compared with classical adenoidectomy because of the precision offered by the improved visual field of the endoscope combined with the extreme manageability of the microdebrider. This allows the surgeon to control the efficient removal of adenoid tissue to the advantage of the patient.
